# Position Estimation in Corridors Along the Coupled Mode of Radiating Cables

**DOI:** 10.3390/s20185064

**Published:** 2020-09-06

**Authors:** Olga Blaszkiewicz, Jaroslaw Sadowski, Jacek Stefanski

**Affiliations:** Faculty of Electronics, Telecommunications and Informatics, Gdansk University of Technology, 80-233 Gdansk, Poland; olga.blaszkiewicz@pg.edu.pl (O.B.); jacek.stefanski@eti.pg.edu.pl (J.S.)

**Keywords:** indoor positioning, indoor navigation, radiating cable, leaky feeder

## Abstract

Radiating cables are mostly used to provide radio communication in tunnels or corridors, but they can also be used to estimate the position of a mobile terminal along the cable. In this paper, a measuring receiver’s position was estimated by measuring the difference in the direct signal’s reception time, which was generated by a transmitter connected to one end of the radiating cable, and the delayed signal retransmitted from another end. During tests, a relatively narrowband (23 MHz) signal was used in the unlicensed band (2.4 GHz) and 50 m long coupled mode radiating cable. The cable was installed along a corridor in the office building. Measurement results used different equipment configurations (i.e., return signal only amplified or amplified and frequency-shifted), which presented possible sources of errors.

## 1. Introduction

One of the most dynamic developing applications of radio communication is the position estimation of people and objects using radio. In outdoor positioning and navigation, a general trend of building versatile solutions has been observed, which may fulfill expectations of different user groups. In such situations, it is not a surprise that global navigation satellite systems (GNSS) become the worldwide standard for commercial and personal positioning. However, as the availability of GNSS services is usually limited to outdoor environments, position estimation inside buildings requires different technologies. Many indoor positioning methods and systems that use different principles of radio wave propagation have already been developed [1] and there is no global agreement on the indoor position estimation technology. Thus, new solutions are still being explored [2].

Position estimation that uses radio techniques in indoor environments often suffers from insufficient accuracy caused by local anomalies in radio wave propagation [3,4]. In many indoor positioning systems that are based on signal level and/or time measurement, a multipath propagation or shadowing via walls, furniture, and humans causes variation in radio frequency (RF) fields. This introduces errors in radio-based position estimations [5,6,7]. Generally speaking, a longer propagation path in the indoor environment results in higher positioning errors [8,9,10], so in many applications it is crucial to deploy the reference nodes close to the positioning system’s area of operation [11]. Unfortunately, this results in either a reduction of the network operation area or an increase in the number of reference nodes required to ensure the correct operation of the positioning system. Instead of using many reference nodes with separate antennas, a radiating cable may be used to provide a better quality positioning signal due to a reduction in propagation path length within a variable environment.

This paper is organized as follows: Section 2 reviews the state-of-the-art research on radiating cable-based positioning. Section 3 describes the principles of radiating cable positioning when measuring differences in reception time of signals travelling in a cable in opposite directions. Section 4 and Section 5 present a measuring stand and the results of position estimation in an indoor building environment, respectively. Section 6 discusses position estimation errors in the proposed solution, while the last section concludes the paper.

## 2. Related Works

As a concept, using radiating cables for positioning is not new. For example, Nishikawa et al. [12] presented a two-dimensional (2D) position of a mobile antenna near a radiating cable and calculated it using a vector network analyzer (VNA), which measured the arrival time for two signal components received via a mobile antenna: a direct signal emitted from a radiating cable and a signal reflected from the open end of the cable. Moschevikin et al. [13] proposed a different approach—i.e., a two-dimensional position estimation of an experimental active terminal—equipped with a transmitter and receiver that simultaneously used round-trip time (RTT) and signal power measurements (e.g., RSS, received signal strength). Although this paper does not summarize position accuracy evaluation, it provide useful information regarding observed propagation of chirp sounding signals emitted by radiating cables. There have been other studies that investigated radio communication quality and RTT distance measurements in both indoor and outdoor environments using a narrowband radiated mode leaky feeder [14].

The RSS measurements were also presented by Engelbrecht et al. in [15]. This publication focused on the construction of a coaxial radiating cable optimized for a system where a cellular phone’s position on the radiating cable (one dimension only) was estimated using two receivers connected to both ends of the cable. Signal’s transmitted via the mobile phone were then coupled to nearby radiating cables and were received by receivers with different power levels. Coupling a signal from a cellular phone to a radiating cable influences both measured signal levels in similar way. However, different power measurement results are caused by longitudinal signal attenuation in the radiating cable which corresponds to terminal position. The same authors described [16,17] a solution based on signal transmission in opposite directions, i.e., two ends of the radiating cable connected to two wireless local area network (WLAN) access points. Signal levels were measured by the terminal located in a long hallway near the radiating cable and compared with a previously prepared radio map. It is considered a variation on the fingerprinting method. Weber et al. [18] presents results and a detailed discussion on how to improve the quality of RSS-based position estimations using a radiating cable. Further, they comment on data smoothing and Kalman filtering.

F. Pereira [19,20] described the simultaneous emission of two signals generated by two transmitters connected to both ends of a radiating cable. He further described how to map signal levels recorded along the cable installed in the tunnels. In addition, Pereira [20] considered the possibility of using a phase-difference measurement in a very high frequency (VHF) band to estimate signal propagation time in a positioning system with a leaky feeder. However, no details, results, nor estimated accuracy were discussed.

Nakamura et al. [21] presents another principal used in this system. The mobile terminal was equipped with a transceiver that amplified and filtered the test signal received from the radiating cable, performed frequency conversion, and retransmission. One end of the radiating cable was connected to the transmitter, which triggered measurements by unmodulated carrier emission. Moreover, the measuring receiver estimated the distance to the mobile device via the round-trip time measurements.

Shirai et al. [22] proposed another method of position estimation using two radiating cables. Both cable ends were connected to four-port receiver. A MUSIC algorithm was used to estimate the impulse response of a MIMO channel. The mobile transmitter was placed near cables and was estimated from delay of the MIMO components in the received signal.

Inomata et al. [23] presented interesting details on passive detection of persons using a pair of radiating cables. In this solution, a sounding signal was radiated from one leaky feeder and received by the second cable, which was parallel to the first one. Target detection was performed when extracting the signal scattered around the moving object. Propagation delay time of the scattered wave was utilized to determine location. Further, they implemented a leaky feeder perimeter intruder detection system. In contrast, the solution presented by Shah et al. [24] detected the presence of an intruder using a radiating cable-based channel state information evaluation without positioning.

Many of the positioning examples mentioned above estimated only one coordinate along the radiating cable, which may be enough for corridors and tunnels. The positioning accuracy reached 0.25 m for a two-dimensional (2D) case using time-based measurements [12], however, was not confirmed in any other publication, even those limited to one-dimensional (1D) position estimation. This may be partially explained by the inclusion of a very wide bandwidth in the sounding signal [12] that reached 1 GHz and used VNA. These factors made one-way propagation delay measurements. A drawback of this method is the necessity to connect the mobile antenna to the VNA via cable; it is not strictly a wireless system. Moreover, a measurement scenario [12] was limited to a cable length of only 5 m, while such short radiating cables were not used in indoor or tunnel radio communications. This paper presents the test results for radiating cables in time-based positioning systems with more realistic signal parameters and configurations. In our research, a general-purpose wideband radiating cable was used for communication systems. But it is worth to mention that special design of radiating cable with non-uniform deployment of slots, proposed by Hassan et al [25] may have improved position estimation quality due to side lobe reduction in cable radiation patterns modelled as linear antenna arrays.

## 3. Principle of Radiating Cable Positioning

A radiating cable is a transmission line (e.g., coaxial, symmetrical) designed to radiate to an external environment a controlled part of the energy of a transmitted signal. In coaxial radiating cables, the emission is caused by an imperfect shielding, i.e., a loosely woven braid or a perforated solid screen. Regarding the different geometry of slots (e.g., shape, spacing), different energy conversion principles are used to model the feeder coupling with the environment [26,27]. The first model assumes that every slot in the cable acts as an elementary magnetic dipole [28]. A resultant electromagnetic (EM) field is a superposition of radiation from every slot. This model is suitable for cables with a non-uniform slot pattern, thus optimizing them for good performance in a narrow frequency band. Such cables are often considered as the “radiating mode”. The cables with closely spaced slots—i.e., the distances between them much shorter than a wavelength—are modeled as a controlled conversion of the energy between the coaxial mode inside the coaxial cable and the one-wire mode between the shielding and environment [29]. These kinds of cables are called the “coupled mode”; their performance depends on the scattering of local fields by nearby objects [30,31]. However, they are frequently used because of their wide bandwidth.

**Figure 1:**
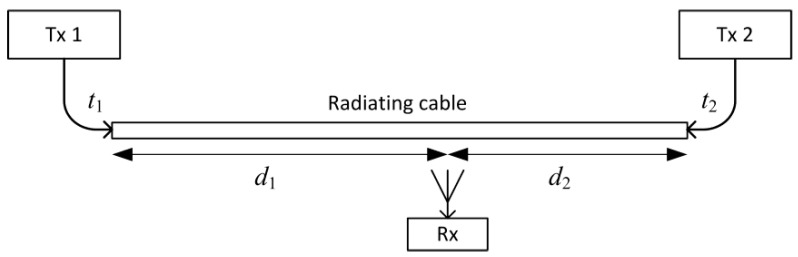
Principle of position estimation along the radiating cable using the time difference of arrival (TDOA) method.

When comparing the indoor radio communication systems with antennas mounted in selected points in buildings, the radiating cable allows for us to achieve more uniform signal power distribution, as the main part of the propagation path is in the cable with predictable longitudinal attenuation. This advantage was used in the positioning method [15,16]. However, a relatively short distance between the measuring device and the nearest part of the radiating cable should also give high repeatability of propagation delay determined by constant signal velocity in the cable and device position along the cable. Assuming that both radiating cable sides are connected to transmitters Tx1 and Tx2 (Figure 1), which transmit the positioning signals at time *t*_1_ and *t*_2,_ respectively (*t*_2_ − *t*_1_ = *T* is known), the measuring receiver Rx receives both signals with a time difference as follows:

(1) $$\Delta t=T+\left(d_{2}-d_{1}\right)\cdot v_{prop},$$

where *v_prop_* is a velocity of the signal in the radiating cable. Therefore, it should be possible to estimate the position of the mobile receiver along the cable by measuring only the difference in positioning signal time of arrival (TDOA). Compared to method presented by Nishikawa et al. [12], this restricts positioning as one dimensional and assumes that signal detection time is caused mostly by emission from the nearest cable section.

The received signal is a superposition of components radiated by a long section of the cable around the nearest point. Due to signal propagation velocity differences in the cable and air, EM wave emission direction is not perpendicular to the cable axis. Thus, position estimation accuracy should depend on the distance of the receiver from the cable. However, the results of our measurements showed that, when using band limited signals, positioning errors caused by these effects may be comparable or even smaller than other error components, such as random errors caused by multipath propagation in corridors when propagation time differences for different paths are lower than signal bandwidth, or errors caused by limited time measurement resolution.

## 4. Measuring Stand

The possibility to estimate the measuring receiver position along the radiating cable was tested in the industrial, scientific, and medical (ISM) band at 2.45 GHz. The signal was generated by a Rohde&Schwarz SMU200 vector signal generator and was modulated using binary phase shift keying (BPSK) modulation with a pseudo random binary sequence (PRBS20) (20 MHz chip rate). The root-raised cosine filter limited bandwidth of transmitted signal to 23.3 MHz (99% of power). The signal level at the generator output was +10 dBm. Therefore, taking into account the coupling loss of the radiating cable, the emission level was far below legal limits. A relatively long PRBS sequence (2^20^ − 1 chips) was necessary to achieve a high processing gain during receiver correlation. This was crucial to extract the test signal from interferences from the IEEE 802.11 networks, which were present in a building where tests were performed.

Test signals were transmitted using a 50 m long RCT4-WBC-1X-RNA coupled mode coaxial radiating cable, which was on the floor of a straight corridor in a faculty building. This eight-story building had reinforced concrete ceilings and columns with brick walls. The dimension of the building was 115 × 12 m and there was a straight corridor along the entire length of the building on every floor. The cross-section of the corridor and the general view are presented in Section 4.3.

The test signals were received with a universal software radio peripheral (USRP) that had a sampling rate equal to 25 MHz and 12-bit conversion. After upsampling 10-times, a time measurement resolution, based on searching for local maxima in the cross-correlation discrete-time function of received signal and PRBS template signal, was equal to 4 ns. During the tests described below, the receiving part was placed on selected points in the corridor (stationary measurements) with an antenna 0.3 m above the floor (0.5 m from the radiating cable). The description of measurement conditions and geometry during the final campaign is presented in Section 4.3.

### 4.1. One Feeder

**Figure 2:**
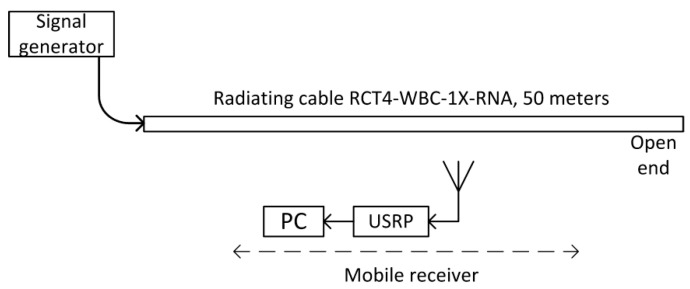
Measuring stand for estimation of position based on the reflection of the test signal from the open end of radiating cable.

The first test verified the possibility of position estimation based on a signal transmitted from one end of the radiating cable and reflected from the open (not terminated) end. Nishikawa et al. [12] successfully presented such a scenario when conducting a test that used VNA. Unfortunately, a limited bandwidth of the pseudo random signal used in our research, made it almost impossible to distinguish between the direct and reflected signal from the radiating cable (Figure 2). Through the direct signal we understand the pseudo random signal from a vector signal generator, transmitted (and delayed) through a radiating cable and radiated into the air in the proximity of the receiver. The reflected signal is the same signal from the vector signal generator, which is transmitted through the radiating cable. It travels length-wise to its unterminated end, reflects from the open end, transmits in a backward direction, and radiates into the air in the proximity of the receiver.

**Figure 3:**
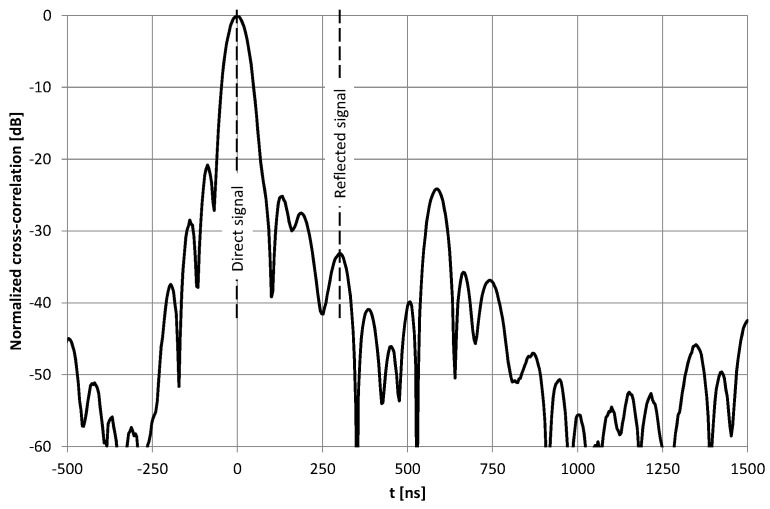
Example of a cross-correlation function of a signal recorded at *d*_1_ = 13.3 m using an unterminated radiating cable.

Near the open end of the cable, the reflected signal was not visible in the correlation function (Figure 3) due to a high level of correlation side lobes. Moreover, in the area closest to the cable end with signal generator, the reflected signal power was attenuated by a long propagation path at the far end of the cable and back. Therefore, automatic detection of the reflected component was not possible. All correlation charts presented in this paper were computed separately using 52.4 ms long fragments of the recorded signals, which was the repetition time of the PRBS20 sequence clocked at a 20 MHz chip rate. No signal filtering or averaging was used, and the recorded signals contained interferences from ISM devices.

As there is only one source of the test signal, the receiver position, *d*_1_, related to the end of the cable connected to signal generator can be expressed by:

(2) $$d_{1}\approx D-\frac{\Delta t}{2}\cdot v_{prop},$$

where Δ*t* is the measured time difference between the direct and reflected signal in the receiver, *D* is the total length of radiating cable, and *v_prop_* is the velocity of the signal propagation in the radiating cable. According to the datasheet, *v_prop_* for cable type RCT4-WBC-1X-RNA is 0.88 c. Although *v_prop_* measurements used the vector network analyzer returned value 0.89 c, we decided to use 0.88 c declared by the producer, because this observed difference in *v_prop_* may cause maximal position error estimation of 0.56 m at *d*_1_ = 0, which was far below the proposed method’s expected accuracy. Equation (2) was accurate only when both signal components received by a measuring device were radiated perpendicular from the section of cable closest to the receiver. Therefore, the time of signal propagation in the air was equal for both components. If the receiver was located close to the cable, the real signal emission at an angle other than perpendicular ([12]) was neglected. The proposed solution was only used for 1D position estimation and in areas close to radiating cable, so possible applications are limited to corridors or tunnels. However, in the real environment, the receiver signal is a superposition of components radiated from some certain part of the leaky feeder. As long as there was sufficient fragment of the radiating cable available in both directions, resulting errors should at least partially cancel each other out. Yet when a mobile receiver was placed near the cable end, the uneven condition of reception of direct and reflected components may cause systematic position estimation errors.

### 4.2. Two Feeders with Amplifier

To improve quality of reception of signal reflected from the end of a cable, an amplifier was used to amplify the signal before sending it back toward the generator.

**Figure 4:**
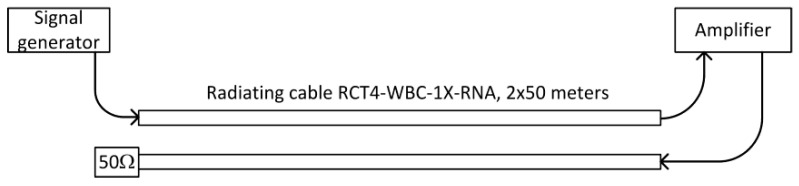
Measuring stand with two radiating cables for a case with return signal amplification.

Two-directional connection of the amplifier to the same cable requires a directional coupler with separation higher than the amplifier gain. This was not available, so we used two parallel radiating cables (Figure 4). Measured coupling loss between two parallel cables, terminated with matched load, placed 20 cm apart, was below -45 dB. To ensure that no oscillations occurred, the amplifier gain was set to 30 dB and the radiating cables were spaced 30–40 cm apart. The second radiating cable was terminated by a matched load. As there was no reflection of the signal from the open end of the radiating cable, the second component of the signal recorded by the measuring receiver was called the return signal. The return signal in this scenario was the pseudo random signal from the vector signal generator connected to the first radiating cable. Then, it was transmitted (and delayed) through the whole length of the first cable, amplified by a wideband amplifier, and delayed in additional coaxial cables. Finally, it is transmitted through the second radiating cable and radiated into the air in the proximity of the receiver.

In this scenario, measuring the receiver position may be estimated as:

(3) $$d_{1}\approx D-\frac{\Delta t-\tau_{c}-\tau_{a}}{2}\cdot v_{prop},$$

where *τ_c_* represents the sum of all additional signal delay in connecting cables and *τ_a_* is a signal delay in an amplifier. All assumptions listed under Equation (2) are still valid.

**Figure 5:**
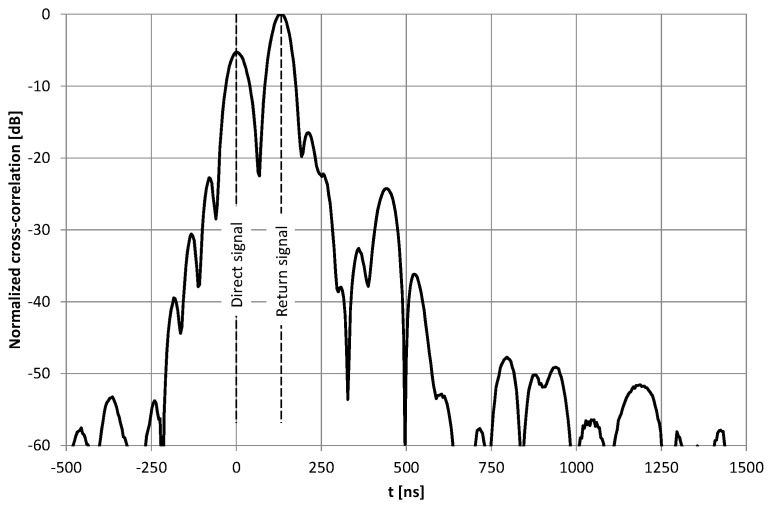
Example of cross-correlation function of signals recorded at *d*_1_ = 39.3 m using two radiating cables with the amplifier.

The amplification and additional delay of the return signal made the detection of the main lobes of the correlation for both components (direct and return) easier. However, in some results the two highest peaks in the correlation function did not correspond to the main lobes of measurement signals. To automatically detect both signals, it is important that near the end of the cables the return signal level may be higher than the level of the direct one (Figure 5).

### 4.3. Two Feeders with Signal Frequency Conversion

**Figure 6:**
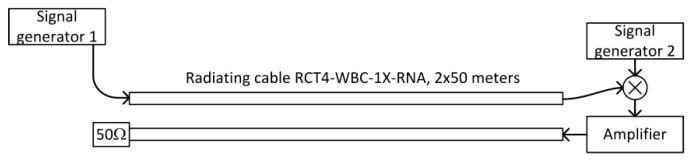
Measuring stand with the frequency conversion of the return signal.

Full separation of the correlation charts for the direct and return signal may be reached with an additional signal delay before amplification using delay times longer than the duration of the unwanted components (side lobes) in the correlation function. It is also possible by modifying the return signal shape or frequency. The simplest method was a frequency conversion that used a balanced mixer and a second signal generator as a heterodyne in the measuring stand (Figure 6). The return signal in this scenario was a frequency-shifted version of the previously defined return signal. It is a pseudo random signal from generator number 1. It was transmitted and delayed in the first radiating cable. Then, it was delayed in an additional coaxial connecting cable and multiplied by a sinusoidal signal from generator number 2. Finally, the return signal was amplified and delayed, then transmitted through the second radiating cable and radiated into the air in the proximity of the measuring receiver. Therefore, both components of the test signal (direct and return) were generated by the first signal generator, but the return signal was additionally mixed with a low-frequency carrier from the second generator. The measuring receiver position was estimated using (3) and by taking into account that *τ_c_* was the sum of the signal delay in cables connecting the first radiating cable with the mixer, the mixer with the amplifier, and the amplifier with the second radiating cable.

**Figure 7:**
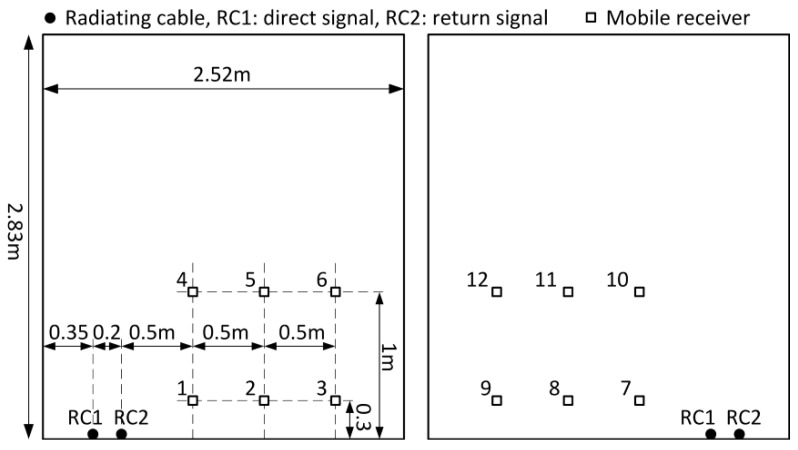
Position of radiating cables and receiving antenna in a corridor in the scenario with two feeders and frequency conversion.

To fully examine the position estimation accuracy in this scenario, measurements were taken in 12 different configurations of the radiating cables (black dots) and the receiving antenna position (squares), presented on a cross-section of the corridor in Figure 7. Firstly, the radiating cables were placed along one wall with cable no. 1 close to the wall. This cable radiated the direct signal. Next, the cables were moved nearer the opposite wall of the corridor so that cable no. 1, radiating the direct signal, was closer to the center of the corridor. The measuring receiver was mounted on a hand cart equipped with a wheel encoder for reference position measurements. Accuracy of this reference data varied from less than 5 cm at the beginning of the measurement trajectory (beginning of radiating cable) up to approximately 20 cm near the end of the cable, due to the measuring wheel slip. The receiving antenna was placed at a height of 0.3 m and 1 m above the floor. The cart speed was from 0.2 to 0.4 m/s along three parallel tracks spaced 0.5 m apart. Additional attenuation of the unconverted signal in the mixer allowed us to reduce the distance between radiating cables to 0.2 m only. In Figure 7, numbers near square marks indicate a measurement series.

**Figure 8:**
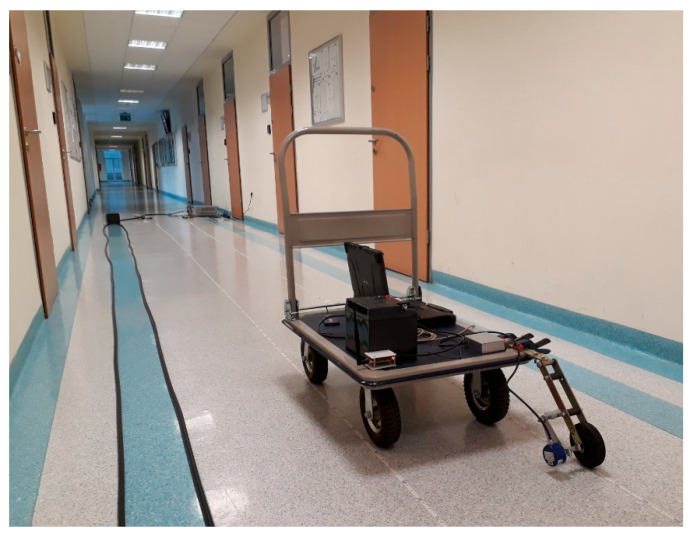
Measurement setup in the scenario with two feeders and frequency conversion.

Figure 8 shows a view of the corridor with radiating cables on the floor and the hand cart with the receiver and the wheel encoder.

The frequency of the signal from the second generator was set to 100 kHz, which was low compared to the transmitted signal’s carrier frequency (2.45 GHz) and the occupied bandwidth (23.3 MHz). Yet when the pseudo random test signal was received by the correlation receiver with an integration time equal to 52.4 ms (2^20^ − 1 times the chip rate), even such a low frequency shift was enough to avoid spectrum despreading of unwanted recorded signal components. Relatively high signal attenuation without conversion in the balanced mixer (over 40 dB) ensured that the signal transmitted by the return cable was composed of only two components at frequencies 2.4499 GHz and 2.4501 GHz, which together occupied a bandwidth of 23.5 MHz. Therefore, the direct and return signals shared approximately the same spectrum. This method does not require wider channel bandwidth in comparison to the previous examples. For such a low-frequency shift, there is no possibility of using a diplexer to separate direct and return signals. In case of using only one radiating cable, the directional coupler is still needed.

**Figure 9:**
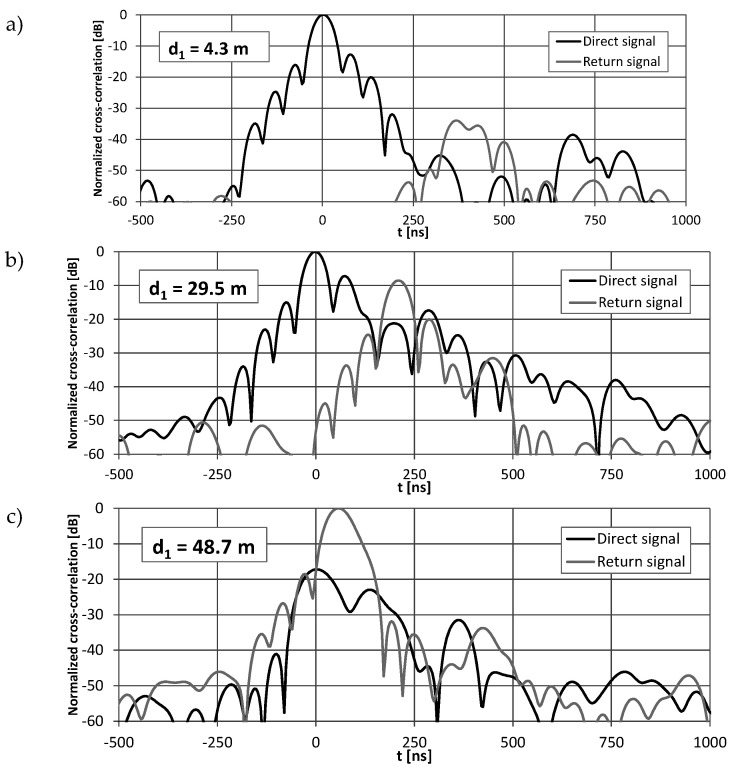
Examples of cross-correlation functions of direct and return signals recorded at *d*_1_ = 4.3 m (**a**), 29.5 m (**b**) and 48.7 m (**c**) using two radiating cables with frequency conversion.

The conversion of the return signal frequency ensured that the reception time of the direct signal was always related to the global maximum in the signal correlation function at a nominal frequency of 2.45 GHz. The reception time of the return signal was obtained from the global maximum of the signal correlation at frequencies 2.45 ± 0.0001 GHz (Figure 9). Therefore, fully automatic detection of both received signal components was trivial. However, examples of the correlation function obtained near the beginning (*d*_1_ = 4.3 m), center (*d*_1_ = 29.5 m), and end of the radiating cable (*d*_1_ = 48.7 m) (Figure 9), shows that only in the center section of the cable shape of the correlation for the direct and return signals is almost the same. Distortion of the correlation function at both ends of the cable, caused by unequal conditions of emission of signals traveling in the opposite direction, may have a significant impact on position estimation accuracy.

## 5. Results of Position Estimation

Due to the low quality of the reflected signal reception in the scenario with an unterminated radiating cable, we could not estimate the mobile receiver’s position. It was caused by overlapping of the reflected signal main lobe with the higher-level side lobes from the direct signal. In this scenario, a wider bandwidth of the test signal would probably improve the discrimination of both components in the received signals. Results obtained from the two other configurations of the transmitting section are more promising.

### 5.1. Two Feeders with Amplifier

**Figure 10:**
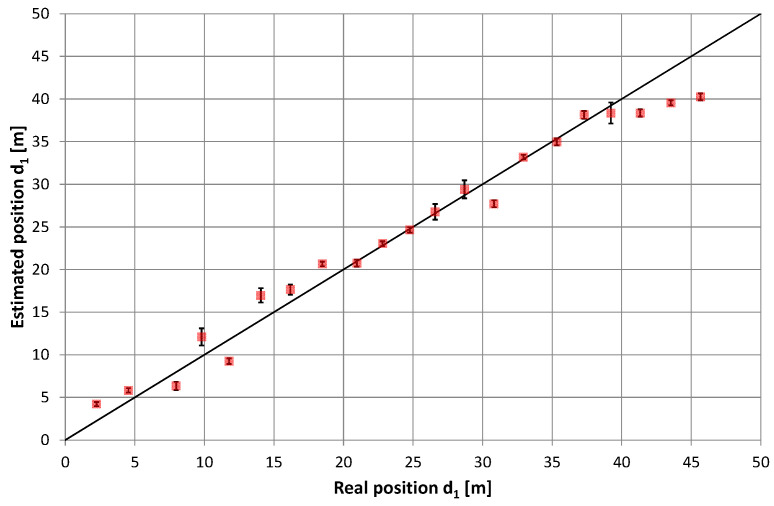
Results of position estimation in the scenario with return signal amplification.

The results of position estimation along two radiating cables with return signal amplification (Figure 10) are median values calculated from 18 repetitions of PRBS signals received during one-second-long signal recordings by the stationary receiver. Error bars in Figure 10 represent the standard deviation of the results. Averaging the results was used to reduce random errors but also caused a reduced update rate to one result per second. If a higher update rate is needed, other methods of data filtering may be used, including the running average and Kalman filtering. Reducing random errors caused by ISM device interference may be achieved after choosing another frequency band.

In general, we saw a high level of repeatability for the obtained results. The standard deviation of position estimation at subsequent measuring points varied from 0.29 m to 1.23 m, but the average error of mean position was several times higher (from −5.41 m to +2.9 m). In addition to the random errors present during measurements, which were characterized by a standard deviation of obtained results, systematic errors were also present in many measurement points and had a higher impact on position estimation accuracy. The best accuracy was found near the center of the radiating cable. High values of mean errors occurred in certain sections of the measured area, which may suggest that it was caused by overlapping of the correlation lobes of the direct and return signals. The systematic shift, observed near both ends of the radiating cable, may be caused by uneven conditions of reception of direct and return signals travelling in opposite directions.

### 5.2. Two Feeders with Signal Frequency Conversion

**Figure 11:**
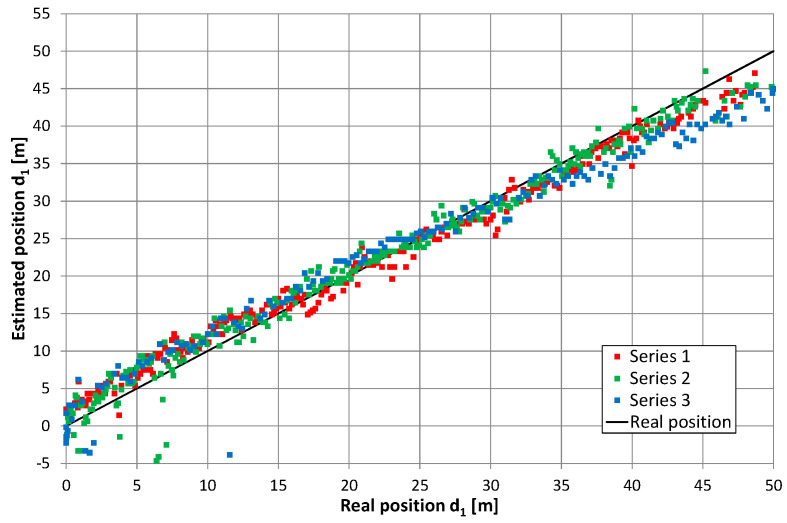
Results of position estimation in the case of return signal frequency conversion; the height of receiving antenna: 0.3 m, series 1–3.

**Figure 12:**
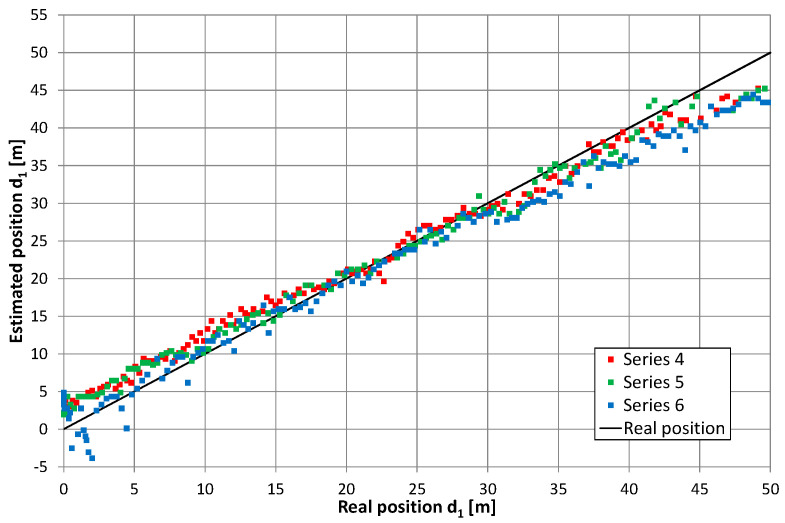
Results of position estimation in the case of return signal frequency conversion; the height of receiving antenna: 1 m, series 4–6.

**Figure 13:**
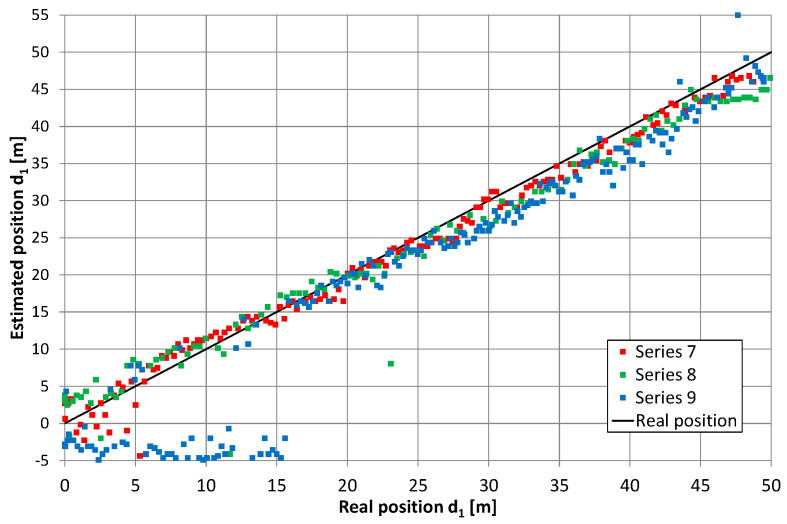
Results of position estimation in the case of return signal frequency conversion; the height of receiving antenna: 0.3 m, series 7–9.

**Figure 14:**
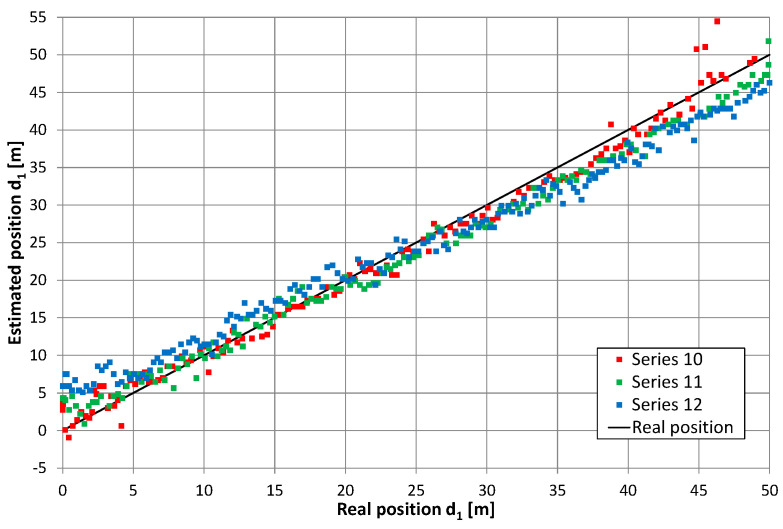
Results of position estimation in the case of return signal frequency conversion; the height of receiving antenna: 1 m, series 10–12.

Converting the return signal frequency should theoretically reduce the mutual impact of the overlapping lobes in the correlation function (i.e., after independent correlation of the direct and return signals). Therefore, the results of the position estimation presented in Figure 11, Figure 12, Figure 13 and Figure 14 reflect the effects caused by the environment and limitations of the proposed positioning method. These measurements were taken on a different day than those presented in Figure 10 (and probably with a slightly different location of the radiating cable in the corridor). Thus, a direct comparison of both charts is not possible. However, there are similarities between results on the charts in Figure 10, Figure 11 and Figure 12, such as systematic errors of position estimation at distances near 31–32 m.

An important difference in the method of measurements presented in this subsection was the receiver’s movement. The estimated position of the receiver was a median value from 18 repetitions of correlation of PRBS signals recorded for one second by a receiver mounted on a moving hand cart. Measurements in motion allowed us to obtain more results in a limited time with an accuracy comparable to those presented in Section 5.1. However, we observed increased dispersion of 18 subsequent results for 1 s long measurements. This was not surprising, as the duration of one correlation of a PRBS20 sequence was comparable and even longer than coherence time for radiating cable communication [32]. Table 1 shows the obtained position estimation accuracy, where $\bar{\varepsilon }$ is the mean value of position error, defined as the difference between estimated and real coordinate *d*_1_, while $\sigma_{\varepsilon }$ is the standard deviation of errors in the final position estimate. The next two variables, included in Table 1, specify short-term data dispersion in a 1-s long measurement. ${\bar{\sigma }}_{s}$ is a mean value of the observed short-term standard deviation in the whole series. $\max\left(\sigma_{s}\right)$ is the maximal value of this parameter in the series. Both the mean value and standard deviation of the position estimation errors were comparable to results presented by other authors for systems with narrowband signals [17].

**Figure 15:**
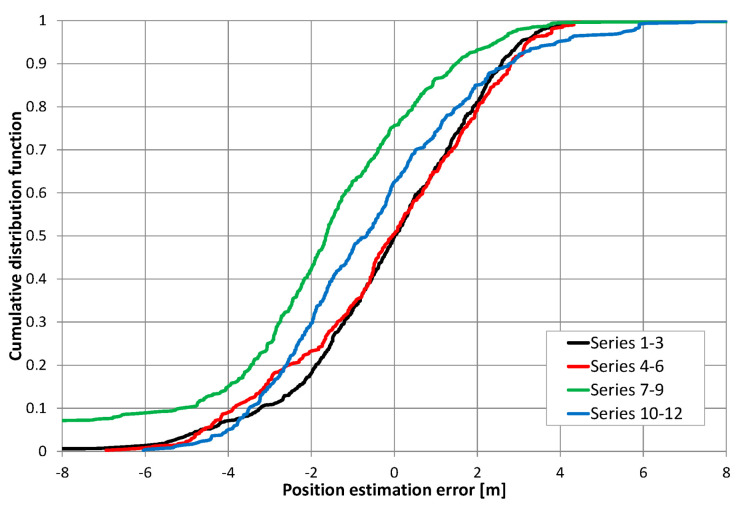
Cumulative distribution of position estimation errors.

Figure 15 presents the cumulative distribution function (CDF) of position estimation error, which is defined as the difference between the estimated and real value of coordinate *d*_1_. In Figure 15, positive quantities indicate position estimates shifted toward the end of the cable with an amplifier, while negative results correspond to position estimates closer to the end of the cable with a signal generator. These charts show almost no difference between results obtained for the receiver antenna at height 0.3 m (series 1–3) and 1 m (series 4–6), as well as no systematic error (CDF equal 0.5 for error value close to zero) when the cable radiating direct signal was closer to the corridor wall. In the second configuration, with the cable radiating direct signal was placed closer to the center of the corridor, the mean error (0.6 m) can be observed for measurements with the receiver antenna at height 1 m (series 10–12). However, when the receiver antenna is 0.3 m above the corridor floor, several measurements returned to the incorrect position of −3.5 m, which is visible in Figure 13 for series no. 9 and in the cumulative distribution function for series 7–9. The almost equal value of position estimation in these incorrect results corresponded to the correct reception of the direct signal and incorrect reception of the return signal traveling through whole length of radiating cable number 2 and then reflected from the end of the cable which was correctly terminated with a 50-ohm load. Therefore, some impedance mismatch or “end effects” [27] probably occurred.

**Figure 16:**
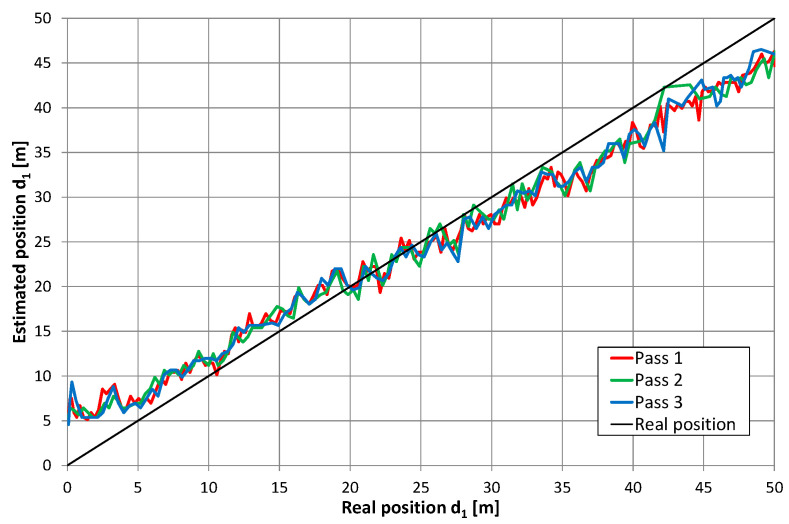
Results of position estimation in three measurement repetitions in series 12; the height of receiving antenna: 1 m.

Although position estimation errors in Figure 11, Figure 12, Figure 13 and Figure 14 were apparently uncorrelated, they were probably caused by inhomogeneous distribution of EM fields inside the corridor. Moreover, they could be caused by a limited measurement setup (e.g., signal bandwidth, measurement resolution). Measurement repetition was performed in exactly the same conditions and showed a high level of error repeatability, which is clearly visible on exemplary charts in Figure 16. Presented results were obtained during three measurement repetitions in series number 12, with the receiver antenna placed 1 m above the corridor floor. Meanwhile, a hand cart moved along the same path with, at most, 5 cm accuracy. In all measurement repetitions, two kinds of errors were distinguished. The first was a systematic shift of position estimates near both ends of the radiating cable. Results obtained in this corridor section indicated that the receiver was closer to the center of the radiating cable; thus, it seemed that these systematic errors may be reduced after evaluating the nonlinear correction function. The second type of error was the repetitive local deviation from the general trend, which at many points exceeded 3 m. This was probably caused by an inhomogeneous building structure and a radiating cable coupling to the building structure. Compensation of these errors may be more difficult and require some kind of fingerprinting method.

We observed high values in position estimation errors for some measurement points, which cannot be explained by corresponding anomalies in the direct and return signals power levels. In general, signal levels along the radiating cables were not stable with random differences exceeding 15 dB. However, no significant changes in power level distribution were found in regions with higher position estimation errors.

In our experiment, the mobile receiver position estimation accuracy was comparable to results presented in the literature. For example, Weber et al. in [16] found position estimation using differences in signal power levels, showing an accuracy of 2.5 m in 50% of all cases and approximately 4.5 m at an 80% threshold level. Pereira et al. [19] had slightly worse results, with 20 m position estimation accuracy at 88% confidence level. They were obtained using GSM and WLAN signals, and they were not dedicated positioning signals. Nakamura et al. [21] showed variable accuracy of distance measurements, from 0.2 m to 8.1 m, with an average error value of 2.4 m. Therefore, it may be concluded that different methods of positioning with radiating cables are comparable for achieving accuracy.

## 6. Discussion on Position Estimation Errors

When evaluating the obtained position estimation accuracy, one should refer to sounding signal parameters, especially in terms of signal bandwidth, which is inversely related to time measurement resolution. For example, ultra-wideband (UWB) indoor positioning systems, based on IEEE 802.15.4 UWB modems using a 499.2 MHz bandwidth, allow for a ranging accuracy of several centimeters [33]. The positioning system based on Nanotron modules, which uses a chirp signal in 2.4 GHz ISM band with a bandwidth of 80 MHz, allows for 1.5 m distance measurement accuracy [34]. However, switching to a 22 MHz bandwidth results in three times worse accuracy. Therefore, the chirp-based solution with a 22 MHz bandwidth may be used as a reference to compare against the proposed solution. In case of the code-division multiple access (CDMA) signal reception in the presence of the Gaussian noise (e.g., AWGN channel, no multipath propagation), theoretical accuracy of tracking the peak of the cross-correlation function may be calculated from Equation (4) [35]:

(4) $$\sigma =\frac{T_{c}}{\sqrt{2\cdot SNR}},$$

where *σ* is the standard deviation of peak time measurement, *T_c_* is chip time (50 ns), and *SNR* is signal-to-noise ratio after spectrum despreading [35]. Exemplary charts, as presented in Section 4, show that during measurement, *SNR* exceeded 40 dB. Therefore, time measurement accuracy limit in the AWGN case reached 0.35 ns, which corresponded to a 0.09 m distance measurement error for radiating cable with *v_prop_* = 0.88 c. However, such good accuracy was not reachable due to multipath propagation, which caused shape degradation of the correlation function’s main peak, which is clearly visible on the first and third charts in Figure 9. Another reference for positioning accuracy evaluation may be the width of the main peak in the cross-correlation function of received signals. Laboratory measurements that used a cable connection between the signal generator and USRP receiver (no multipath or external interferences) gave a main peak width equal to 48 ns at −3 dB, which corresponded to a distance of 12.7 m. However, the time measurement resolution 4 ns, defined by the receiver’s sampling frequency (25 MHz) and a 10-times up-sampling rate, corresponded to 1.06 m of a one-way distance measurement resolution. Therefore, we achieved position estimation accuracy close to the receiver’s measurement resolution.

We mitigated time measurement uncertainty during tests by using the following tools: the accuracy of frequency standard in signal generators (at most 10^−7^), the accuracy of a reference oscillator in the USRP receiver (at most 2 × 10^−6^), the accuracy of the signal delay in the amplifier and connecting cables (±0.2 ns), and propagation speed *v_prop_*. The impact of all other sources of uncertainty were several orders of magnitude smaller than the observed errors caused by inhomogeneous emissions of radio signals and cables that effect the environment.

The wide width of the main peak in the correlation function equaled 48 ns and was strictly connected to the limited possibility of separating the multipath components in the receiver. There was no possibility of investigating multipath phenomena using signals recorded by the setup presented in this paper. Signals received in the shorter period overlap, thus distorting the shape of correlation function and causing errors when detecting signals’ timing based on peak tracking. However, in typical indoor or outdoor radio positioning systems, based on radio signal propagation in the air, time measurement quality may be improved with a leading-edge detector because the multipath components reach the receiving antenna after the signal travels in a straight (and short) path. However, in a positioning system based on a radiating cable, unwanted multipath components may be received before a signal radiated perpendicular to the cable, i.e., signal emissions with high power from a section of cable not close to a point near the receiver. This signal can reach the receiver after time of propagation in the air (with speed c), even if earlier than wanted component which has to travel through the radiating cable (with speed *v_prop_* lower than c). Therefore, the advantage of slope detection over peak detection in positioning systems using radiating cables is questionable.

**Figure 17:**
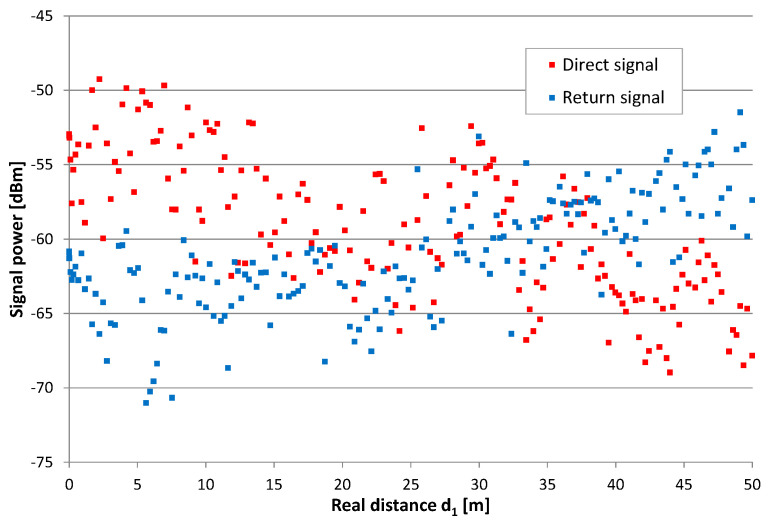
Exemplary values of the power level of direct and return signal, series 12.

Results from all measurements clearly shows regularity. The best accuracy was available near the center of radiating cables, while position estimates obtained near both ends of the cable were systematically shifted toward the center. This effect was not caused by the wrong value of signal propagation speed in cable *v_prop_*, because incorrect *v_prop_* in the setup (Figure 4 and Figure 6) would cause the best match near the end of the radiating cable and increase error in the region closer to the beginning of the cable. These systematic errors are likely caused by unequal radiation conditions when signals travel in the opposite direction in finite-length radiating cables. The direction of radiation of the EM field is skewed to the direction of signal propagation in the cable [12,29]. Additionally, the received signal is always superposition of components radiated from some section of the cable. Thus, the measurements taken near the end of the cable may correspond to different EM field distribution comparing to center section of the cable. In general, electromagnetic field emissions from radiating cables was not uniform [36,37,38], causing time measurements errors and a large variations of instantaneous received signal power values that exceeded 12 dB. This is visible on the power chart presented in Figure 17, as a random deviation from linear trend of power drop which was caused by leaky feeder longitudinal attenuation.

A general trend of power level changes in Figure 17 is similar to the systematic character of the obtained position estimation error. The correction of position had a linear approximation of difference in the power of direct (*P_dir_*) and return (*P_ret_*) signals (in decibels) when using Equation (5):

(5) $$d_{1}^{\prime }=d_{1}-0.298\cdot \left(P_{dir}-P_{ret}\right)+0.95$$

Only partially improved position estimation accuracy came from reducing the mean error $\bar{\varepsilon }$ to zero and standard deviation $\sigma_{\varepsilon }$ from series 12 to 2.37 m (from 3.23 m without correction). It may be expected that a large dispersion of instantaneous power from the received signals was caused by a multipath fading phenomena and standing waves, which may have increased local dispersion of the position estimation results even when the systematic position shifted near both ends of the cable was reduced. It was not possible to remove the fading effect from the measured power levels without spatial data averaging over a long path, exceeding tens of wavelengths. Thus, data correction by using signal power levels is of limited use. Another method of data correction was evaluated using the least squares linear model of the position errors, which may be summarized as follows:

(6) $$d_{1}^{\prime }=d_{1}+0.233\cdot d_{1}-5.28$$

**Figure 18:**
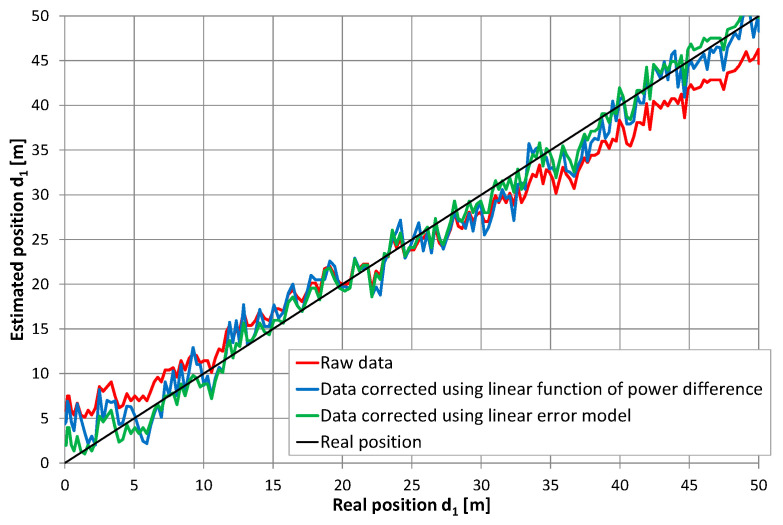
Results of position estimation in series 12 with correction based on the difference in direct and return signal power levels and corrections based on the linear model’s position errors.

Such a simple correction allowed us to reduce a standard deviation to 1.46 m, giving better results than corrections based on differences in the received signal power. Both data correction methods are presented in Figure 18 using blue (correction based on the signal power levels) and green (correction based on a linear error model) lines, respectively. In general, both methods reduced systematic position shifts near the ends of the radiating cable. Both gave the mean value of the position error close to zero. Differences between them were visible not only for standard deviation value but also on local result variation (Figure 18). Maximal values of uncorrected errors in series 12 were −6.04 m and +7.29 m. Corrections based on signal power levels reduced maximal error values to −5.24 m and +6.71 m, while corrections based on the simple linear model gave maximal errors of −4.13 m and +3.75 m. Therefore, both data correction methods are able to reduce systematic position shift, which is visible in the raw data near both ends of the radiating cable. Unfortunately, reducing local anomalies in position estimates using received signal power levels was unsuccessful, as this method results in higher errors.

It is difficult to explain high values of errors visible on some charts in the 17–20 m and 30–32 m regions. The whole corridor was free from obstacles during measurements. The first region was situated near a staircase. The second region was not connected with any changes in the geometry of the corridor, but it turned out that, in this region, there was a boundary between two structural sections of the building with thick reinforced walls on both sides of the corridor instead of brick walls. Taking into account that the radiating cable’s signal emissions were connected with scattered EM fields, anomalies in position estimation may be caused by inhomogeneous geometry and building structure. Unfortunately, we could not find any area with a strictly homogeneous structure, because even in the outdoor environment, some underground infrastructure was always present (e.g., pipes, cables).

## 7. Conclusions

Estimating the receiver position along the radiating cable using time-difference measurements of a relatively narrowband (23 MHz) signal is possible and very promising. The obtained results can form a basis for developing radiolocation systems in corridors or tunnels where radiating cables are already installed, without the need to deploy a dense network of reference nodes required in UWB-based solutions. For a full scope of the possibilities presented by the proposed solution, extended measurements should be performed in different parts of the corridor or with different deployments of the radiating cable, such as under the ceiling and separated from any conductive elements. However, the proposed solution only allows for one dimensional position estimation in a limited area near the radiating cable, which may be assessed as the biggest disadvantage of radiating cable-based positioning.

## Figures and Tables

**Table 1:** Evaluation of position estimation accuracy.

Series	$\bar{\varepsilon }$ [m]	$\sigma_{\varepsilon }$ [m]	${\bar{\sigma }}_{s}$ [m]	$\max\left(\sigma_{s}\right)$ [m]
1	−0.02	1.95	1.46	3.74
2	0.05	2.17	2.3	8.25
3	−0.7	3.18	1.96	4.72
4	0.64	2.09	2.07	15.7
5	0.17	2.06	1.69	7.82
6	−1.32	2.61	2.27	5.98
7	−0.62	1.71	2.79	9.95
8	−1.02	2.93	3.47	10.7
9	−3.77	7.76	3.63	11.4
10	−0.17	1.71	2.57	7.57
11	−0.92	1.89	2.47	7.6
12	−0.39	3.23	1.73	3.48

## References

[1] Liu H., Darabi H., Banerjee P., Liu J. (2007). Survey of Wireless Indoor Positioning Techniques and Systems. IEEE Trans. Syst. Man Cybern. Part C Appl. Rev..

[2] Laoudias C., Moreira A.J., Kim S., Lee S., Wirola L., Fischione C. (2018). A Survey of Enabling Technologies for Network Localization, Tracking, and Navigation. IEEE Commun. Surv. Tutori..

[3] Müller P., Raitoharju M., Piché R. A Field Test of Parametric WLAN-Fingerprint-Positioning Methods. Proceedings of the 17th International Conference on Information Fusion.

[4] Wang W., Marelli D., Fu M. (2020). Fingerprinting-Based Indoor Localization Using Interpolated Preprocessed CSI Phases and Bayesian Tracking. Sensors.

[5] Zayets A., Steinbach E. Robust WiFi-based indoor localization using multipath component analysis. Proceedings of the 2017 International Conference on Indoor Positioning and Indoor Navigation (IPIN).

[6] Xue W., Qiu W., Hua X., Yu K. (2017). Improved Wi-Fi RSSI Measurement for Indoor Localization. IEEE Sens. J..

[7] Phutcharoen K., Chamchoy M., Supanakoon P. Accuracy Study of Indoor Positioning with Bluetooth Low Energy Beacons. Proceedings of the 2020 Joint International Conference on Digital Arts, Media and Technology with ECTI Northern Section Conference on Electrical, Electronics, Computer and Telecommunications Engineering (ECTI DAMT & NCON).

[8] Di Pietra V., Dabove P., Piras M., Lingua A. Evaluation of positioning and ranging errors for UWB indoor applications. Proceedings of the 2019 International Conference on Indoor Positioning and Indoor Navigation (IPIN).

[9] Barua B., Kandil N., Hakem N. On performance study of TWR UWB ranging in underground mine. Proceedings of the 2018 Sixth International Conference on Digital Information, Networking, and Wireless Communications (DINWC).

[10] Minghui Z., Huiqing Z. Research on model of indoor distance measurement based on receiving signal strength. Proceedings of the 2010 International Conference on Computer Design and Applications.

[11] Ji M., Kim J., Jeon J., Cho Y., Myungin J. Analysis of positioning accuracy corresponding to the number of BLE beacons in indoor positioning system. Proceedings of the 2015 17th International Conference on Advanced Communication Technology (ICACT).

[12] Nishikawa K.-I., Higashino T., Tsukamoto K., Komaki S. (2008). A new position detection method using leaky coaxial cable. IEICE Electron. Express.

[13] Moschevikin A., Serezhina M., Sikora A. On the possibility to use leaky feeders for positioning in chirp spread spectrum technologies. Proceedings of the 2014 2nd International Symposium on Wireless Systems within the Conferences on Intelligent Data Acquisition and Advanced Computing Systems.

[14] Serezhina M., Moschevikin A., Evmenchikov R., Sikora A. Using radiating cable for time-of-flight CSS measurements indoors and outdoors. Proceedings of the 2015 IEEE 8th International Conference on Intelligent Data Acquisition and Advanced Computing Systems: Technology and Applications (IDAACS).

[15] Engelbrecht J., Collmann R., Birkel U., Weber M. Methodical leaky feeder design for indoor positioning considering multipath environments. Proceedings of the 2010 IEEE Radio and Wireless Symposium.

[16] Ott A.T., Shalaby M., Siart U., Kaliyaperumal E., Eibert T.F., Engelbrecht J., Collmann R. (2011). Performance analysis of a low cost wireless indoor positioning system with distributed antennas. Adv. Radio Sci..

[17] Birkel U., Weber M., Collmann R. Radiating Cable for indoor localization using UMTS. Proceedings of the 2012 Ubiquitous Positioning, Indoor Navigation and Location Based Services.

[18] Weber M., Birkel U., Collmann R., Engelbrecht J. Comparison of various methods for indoor RF fingerprinting using leaky feeder cable. Proceedings of the 2010 7th Workshop on Positioning, Navigation and Communication.

[19] Pereira F., Theis C., Moreira A., Ricardo M. Multi-technology RF fingerprinting with leaky-feeder in underground tunnels. Proceedings of the 2012 International Conference on Indoor Positioning and Indoor Navigation.

[20] Pereira F. (2016). Positioning Systems for Underground Tunnel Environments. Ph.D. Thesis.

[21] Nakamura M., Takagi H., Terashima J., Einaga K., Nishikawa T., Moriyama N., Wasaki K. Development of a Simple Multiple-Position Identifying System with a Long Range Multiband Leaky Coaxial Cable for Rescue Operations in Tunnels or Passages in Underground Facilities. Proceedings of the Asia-Pacific Microwave Conference.

[22] Shirai K., Higashino T., Okada M. An Experimental Investigation of the MUSIC-based Wireless Position Location using LCX antenna at 5GHz band. Proceedings of the 2019 19th International Symposium on Communications and Information Technologies.

[23] Inomata K., Hirai T., Yamaguchi Y., Yamada H. (2008). Two-Dimensional Target Location Estimation Technique Using Leaky Coaxial Cables. IEICE Trans. Commun..

[24] Shah S.I., Shah S.Y., Shah S.A. Intrusion Detection through Leaky Wave Cable in Conjunction with Channel State Information. Proceedings of the 2019 UK/China Emerging Technologies.

[25] Hassan N., Fernando X.N. Reduced side lobe MM-wave leaky feeder transceiver by slot space optimization. Proceedings of the Global Symposium on Millimeter-Waves.

[26] Wang J.H., Mei K.K. (2001). Theory and Analysis of Leaky Coaxial Cables with Periodic Slots. IEEE Trans. Antennas Propag..

[27] Martin D.J.R. (1975). A general study of the leaky-feeder principle. Radio Electron. Eng..

[28] Cao H., Zhang Y.P. Radio Propagation along a Radiated Mode Leaky Coaxial Cable in Tunnels. Proceedings of the 1999 Asia Pacific Microwave Conference.

[29] Guo Y.C., Zhang Y.P. Radio Propagation along a Coupled Mode Leaky Coaxial Cable in Tunnels. Proceedings of the 1999 Asia Pacific Microwave Conference.

[30] Aragon-Zavala A. (2017). Indoor Wireless Communications.

[31] Delogne P.P., Deryck L. (1980). Underground Use of a Coaxial Cable with Leaky Sections. IRE Trans. Antennas Propag..

[32] Pan Y.-T., Liu X., Zheng G.-Z., Guan K. (2018). Temporal Autocorrelation of Small-Scale Fading Using Leaky Coaxial Cable in Confined Space. IEEE Wirel. Commun. Lett..

[33] Malajner M., Planinsic P., Gleich D. UWB ranging accuracy. Proceedings of the 2015 International Conference on Systems, Signals and Image Processing.

[34] Pivato P., Dalpez S., Macii D. Performance Evaluation of Chirp Spread Spectrum Ranging for Indoor Embedded Navigation Systems. Proceedings of the 7th IEEE International Symposium on Industrial Embedded Systems.

[35] Yu K., Sharp I., Guo J. (2009). Ground-Based Wireless Positioning.

[36] Santos V., da Fonseca F., de Matos L., Meza W., Siqueira G. Indoor Signal Coverage of a Leaky Feeder Cable. Proceedings of the IEEE MTT-S International Microwave & Optoelectronics Conference.

[37] Buffi A., Nepa P., Tellini B. Measurement System with Leaky Coaxial Cables operating as Distributed Antennas for UHF-RFID Readers. Proceedings of the 2017 IEEE International Workshop on Measurement and Networking.

[38] Jiang J., Wang L., Wang G. Leaky Coaxial Cable for Near-field UHF RFID Applications. Proceedings of the 2017 Sixth Asia-Pacific Conference on Antennas and Propagation.

